# Ischemia-Reperfusion Injury Reduces Kidney Folate Transporter Expression and Plasma Folate Levels

**DOI:** 10.3389/fimmu.2021.678914

**Published:** 2021-06-04

**Authors:** Cheng Yang, Charith U. B. Wijerathne, Guo-wei Tu, Connie W. H. Woo, Yaw L. Siow, Susara Madduma Hewage, Kathy K. W. Au-Yeung, Tongyu Zhu, Karmin O

**Affiliations:** ^1^Department of Urology, Zhongshan Hospital, Fudan University, Shanghai, China; ^2^Key Laboratory of Organ Transplantation, Shanghai, China; ^3^St. Boniface Hospital Research Centre, Winnipeg, MB, Canada; ^4^Department of Animal Science, University of Manitoba, Winnipeg, MB, Canada; ^5^Department of Critical Care Medicine, Zhongshan Hospital, Fudan University, Shanghai, China; ^6^Department of Pharmacology and Pharmacy, University of Hong Kong, Hong Kong, China; ^7^Agriculture and Agri Food Canada, Winnipeg, MB, Canada; ^8^Department of Physiology and Pathophysiology, University of Manitoba, Winnipeg, MB, Canada

**Keywords:** acute kidney injury, ischemia, folate, folate transporters, tubular cells

## Abstract

Acute or chronic kidney disease can cause micronutrient deficiency. Patients with end-stage renal disease, kidney transplantation or on dialysis have reduced circulating levels of folate, an essential B vitamin. However, the molecular mechanism is not well understood. Reabsorption of folate in renal proximal tubules through folate transporters is an important process to prevent urinary loss of folate. The present study investigated the impact of acute kidney injury (AKI) on folate transporter expression and the underlying mechanism. AKI was induced in Sprague-Dawley rats that were subjected to kidney ischemia (45 min)-reperfusion (24 h). Both male and female rats displayed kidney injury and low plasma folate levels compared with sham-operated rats. The plasma folate levels were inversely correlated to plasma creatinine levels. There was a significant increase in neutrophil gelatinase-associated lipocalin (NGAL) and IL-6 mRNA expression in the kidneys of rats with ischemia-reperfusion, indicating kidney injury and increased inflammatory cytokine expression. Ischemia-reperfusion decreased mRNA and protein expression of folate transporters including folate receptor 1 (FOLR1) and reduced folate carrier (RFC); and inhibited transcription factor Sp1/DNA binding activity in the kidneys. Simulated ischemia-reperfusion through hypoxia-reoxygenation or Sp1 siRNA transfection in human proximal tubular cells inhibited folate transporter expression and reduced intracellular folate levels. These results suggest that ischemia-reperfusion injury downregulates renal folate transporter expression and decreases folate uptake by tubular cells, which may contribute to low folate status in AKI. In conclusion, ischemia-reperfusion injury can downregulate Sp1 mediated-folate transporter expression in tubular cells, which may reduce folate reabsorption and lead to low folate status.

## Introduction

Acute kidney injury (AKI) is characterized by a rapid decline of kidney function over a short period of time and often progresses to chronic kidney disease (CKD). Despite improvements in renal replacement therapy, AKI is associated with a high morbidity and mortality (1). AKI occurs in patients with kidney transplantation, cardiac surgery, sepsis or critical illness (2–4). The principal function of the kidneys is to maintain body fluid volume, electrolyte and acid-base balance as well as regulating the excretion of wastes and reabsorption of nutrients or metabolic products (5, 6). Micronutrient deficiency is common in patients with kidney disease and malnutrition is an independent predictor of AKI mortality (7). Patients with CKD or end-stage renal disease have reduced circulating folate levels despite adequate dietary intake or receiving renal replacement therapy (8–13). A recent study reported a decreased circulating folate level with an elevated homocysteine level in adolescents and young adults undergoing kidney transplantation (14).

Folate is an essential micronutrient (vitamin B9) that participates in methylation reactions, nucleic acid synthesis and sulfur-containing amino acid metabolism. Folic acid, a synthetic form of folate, is used as a supplement or in fortified foods (15, 16). General populations in countries with mandatory fortification policies have achieved adequate intake of folate (15). The kidneys play an important role in maintaining folate homeostasis in the body through glomerular filtration and tubular reabsorption process. Folate in the circulation, mainly in the form of 5-methyltetrahydrofolate (5-MTHF), is freely filtered in glomeruli. Much of the filtered folate is reabsorbed in the proximal tubules. This reabsorption process is an important mechanism to prevent urinary loss of folate (17). Folate receptor alpha or folate receptor 1 (FOLR1) is the major transporter for folate reabsorption in the kidneys. FOLR1 is located on the apical surface of proximal tubular cells. Folate is transported by FOLR1 from the lumen of tubules into tubular cells *via* receptor-mediated endocytosis (17–19). The promoter region of FOLR1 gene contains transcription factor Sp1 binding sites (20–22). Tubular cells also express other folate transporters. The reduced folate carrier (RFC) is located in the basolateral membrane of proximal tubular cells. RFC transports folate into the peritubular capillaries through a bidirectional anion-exchange mechanism (23). Proton-coupled folate transporter (PCFT), mainly responsible for dietary folate absorption in the intestine, is also present in the kidneys (24).

Ischemia-reperfusion is one of the most common causes for AKI (6). Proximal tubular cells are more susceptible to ischemia-reperfusion injury (25, 26). We have observed tubular damage and declining kidney function in animal models with ischemia-reperfusion induced AKI (27–34). The expression of folate transporters is closely linked to folate status and its biological function. Although low serum folate levels have been reported in patients with CKD, kidney transplant recipients or on dialysis, limited information is available regarding the impact of AKI on folate transporter expression and folate status. In the present study, we investigated (1) the impact of ischemia-reperfusion on renal folate transporter expression and folate status in rats; and (2) the potential mechanism of altered folate transporter expression in human proximal tubular cells.

## Materials and Methods

### Induction of Kidney Ischemia-Reperfusion in Rats

Sprague-Dawley rats (270-300 g, male and female) were randomly divided into two groups that were subjected to kidney ischemia-reperfusion or sham-operation. Kidney ischemia was induced by clamping the left renal pedicle with a non-traumatic vascular clamp for 45 min. At the end of ischemia, the clamp was removed to allow blood flow to the left kidney (reperfusion) and right nephrectomy was performed as described in our previous studies (27–31). Rats were sacrificed 24 h after reperfusion. In the sham-operated group (control), rats were subjected to the same surgical procedure but without inducing kidney ischemia and were sacrificed at the corresponding time point. All procedures were performed in accordance with the Guide to the Care and Use of Experimental Animals published by the Canadian Council on Animal Care and approved by the University of Manitoba Protocol Management and Review Committee. Plasma creatinine was measured using the Cobas C111 analyzer (Roche, Laval, Canada). Total folate in the plasma and proximal tubular cells was measured by using the *L. rhamnosus* coated microtitre plates according to the manufacturer’s instructions provided in the Folic acid Vitamin B9 Microbiological Test Kit (ALPCO, Salem, NH).

### Histological and Immunohistochemical Staining

Kidneys were excised and immersion-fixed in 10% neutral-buffered formalin, embedded in paraffin and sectioned at 5 µm. Sections were stained with hematoxylin and eosin (H&E) to examine histological changes in the kidney (28, 35). For immunohistochemical staining, sequential sections (10 µm) were prepared from cryofixed kidney. Sections were incubated with primary antibodies, rabbit anti-FOLR1 monoclonal antibody (1:100 Abcam, Cambridge, MA) or rabbit anti-SLC19A1 (RFC) (1:100, Abcam) monoclonal antibody and then with biotinylated goat anti-rabbit IgG (1:200, Dako, Glostrup, Denmark) secondary antibody followed by incubation with streptavidin-horse radish peroxidase (HRP) conjugate (Zymed Laboratories, Inc., San Francisco, CA). For a negative control, non-specific rabbit IgG was used as primary antibodies. The images from H&E and immunohistochemical staining were captured by using Olympus BX43 light microscope equipped with an Olympus Q-Color3 camera.

### Induction of Hypoxia-Reoxygenation in Tubular Cells

Human kidney proximal tubular cell line (HK-2) was purchased from the American Type Culture Collection (ATCC, Manassas, VA). Cells were cultured at 37°C in a normal atmosphere of 95% air and 5% CO_2_ in keratinocyte-serum free medium supplemented with human recombinant epidermal growth factor and bovine pituitary extract (Life Technologies, Carlsbad, CA). Hypoxia-reoxygenation was induced in tubular cells to simulate ischemia-reperfusion injury as described in previous studies (30, 31, 36). In brief, hypoxia was induced in tubular cells by incubation for 2 h in a modified Krebs buffer (137 mM NaCl sodium chloride, 15.8 mM KCl potassium chloride, 0.49 mM MgCl_2_ magnesium chloride, 0.9 mM CaCl_2_ calcium chloride, 4 mM HEPES) supplemented with 10 mM 2-deoxyglucose, 20 mM sodium lactate, 12 mM KCl potassium chloride and 1 mM sodium dithionite (pH 6.4) in a hypoxia chamber (Billups-Rothenberg, Inc., Del Mar, CA) containing 95% N_2_/5% CO_2_ at 37°C. After 2 h hypoxia, the modified Krebs buffer was replaced with keratinocyte-serum free medium and cells were cultured for 48 h to 72 h in 95% air and 5% CO_2_. Cells without induction of hypoxia were used as a control.

### Measurement of mRNA Expression

Total RNA was isolated from kidneys or proximal tubular cells by using TRIzol reagent (Thermo Fisher Scientific, Waltham, MA) and 1-2 µg was converted to cDNA through reverse transcription. The real-time PCR reaction mixture consisted of 2 µL of cDNA product, 0.4 µM of 5’ and 3’ primers and iQ-SYBR green supermix reagent (Bio-Rad, Mississauga, Canada). The mRNA expression of folate receptor-1 (FOLR1), reduced folate carrier (RFC), proton-coupled folate transporter (PCFT), specificity protein-1 (Sp1), neutrophil gelatinase-associated lipocalin (NGAL) and interleukin-6 (IL-6) was determined by using a StepOnePlus™ Real-Time PCR System (Life Technologies). The mRNA expression was calculated using the threshold cycle (2−ΔΔCT) method with β-actin as an internal reference. The primer sequences used in this study are listed in **Table 1**.

**Table 1:** Gene primer sequences of human and rat used for real-time PCR.

Target gene	Forward Primer (5’-3’)	Reverse Primer (5’-3’)	Size (bp)
**Human**			
FOLR1	ACTGGACTTCAGGGTTTAACAAG	GTAGGAGTGAGTCCAGATTTCATT	110
RFC	ATGGTGCCCTCCAGCCCAGCGGTG	TCACTGGTTCACATTCTGAACACC	1780
PCFT	ATGCAGCTTTCTGCTTTGGT	GGAGCCACATAGAGCTGGAC	100
Sp1	GCCTCCAGACCATTAACCTCAG	TCATGTATTCCATCACCACCAG	148
IL-6	AGGAGACTTGCCTGGTGAAA	GTCAGGGGTGGTTATTGCAT	182
β-actin	AGATCAAGATCATTGCTCCTCCT	GATCCACATCTGCTGGAAGG	95
**Rat**			
FOLR1	CGGAGACAAGGGTAGGTGTG	TTGAGAAGTTCGGTCCTGGC	166
RFC	CATGCTAAGCGAACTGGTGA	TTTTCCACAGGACATGGACA	122
PCFT	AAGCCAGTTATGGGCAACAC	GGATAGGCTGTGGTCAAGGA	300
Sp1	GGCTACCCCTACCTCAAAGG	CACAACATACTGCCCACCAG	103
NGAL	GATCAGAACATTCGTTCCAA	TTGCACATCGTAGCTCTGTA	91
IL-6	CCGGAGAGGAGACTTCACAG	ACAGTGCATCATCGCTGTTC	161
β-actin	ACAACCTTCTTGCAGCTCCTC	GACCCATACCCACCATCACA	198

FOLR1, folate receptor 1; RFC, reduced folate carrier; PCFT, Proton-coupled folate transporter; Sp1, Specificity protein-1; NGAL, neutrophil gelatinase-associated lipocalin; IL-6, interleukin-6.

### Western Immunoblotting Analysis

The folate receptor proteins (FOLR1, RFC and PCFT) were determined by Western immunoblotting analysis. Proteins (10-50 µg) isolated from kidneys or proximal tubular cells were separated in SDS-PAGE (10-12%) and subsequently transferred to a nitrocellulose membrane. The membrane was probed with primary antibodies, rabbit anti-FOLR1 monoclonal antibody (1:1000, Abcam), rabbit anti-SLC19A1 monoclonal antibody (RFC, 1:1000, Abcam) or rabbit anti-HCP1 (PCFT) monoclonal antibody (1:1000, Abcam) followed by HRP conjugated anti-rabbit IgG secondary antibody (1:2000, Cell Signaling Technology, Danvers, MA). For Sp1 protein, the membrane was probed by goat anti-Sp1 polyclonal antibody (1:1000, Santa Cruz Biotechnology, CA, USA) followed by HRP conjugated anti-goat IgG secondary antibody (1:1000, Thermo Fisher Scientific). Equal protein loading of each sample was confirmed by probing the nitrocellulose membrane with rabbit anti-β-actin monoclonal antibody (1:1000, Cell Signaling Technology). The protein bands were visualized by using Luminata Crescendo chemiluminescent HRP detection reagent (Millipore Ltd, Etobicoke, Canada).

### Electrophoretic Mobility Shift Assay

Nuclear proteins were isolated from kidney tissue and the Sp1/DNA binding activity was measured by electrophoretic mobility shift assay (EMSA). In brief, oligonucleotides were biotin-labelled by using the Biotin 3’ End DNA labelling kit (Thermo Fisher Scientific). The sequence of the Sp1 oligonucleotide probe was 5´-CTGGGCTGGGC-3´. The EMSA was performed by using the LightShift Chemiluminescent EMSA Kit (Thermo Fisher Scientific).

### Cell transfection With Sp1 Small Interfering RNAs (siRNAs)

Proximal tubular cells were transfected with human Sp1 siRNA duplex oligoribonucleotides (25 pmol, Stealth RNAi™, Invitrogen, Carlsbad, CA) by using Lipofectamine 2000 transfection reagent (Invitrogen) according to the manufacturer’s instructions. As a control, cells were transfected with Stealth™ RNA negative control (Invitrogen) consisting of a scrambled sequence that was unable to inhibit gene expression. At 72 h after transfection, cells were harvested and total mRNAs were prepared. The Sp1 mRNA and FOLR1 mRNA were determined. In another set of experiments, intracellular folate content was measured 72 h after transfection.

### Statistical Analysis

The results were analyzed by using two-tailed Student’s t-test or Pearson correlation test. The data were presented as mean ± standard error (SE). A *p* value less than 0.05 was considered as statistically significant.

## Results

### Ischemia-Reperfusion Impaired Kidney Function and Reduced Circulating Folate Levels in Rats

**Figure 1:**
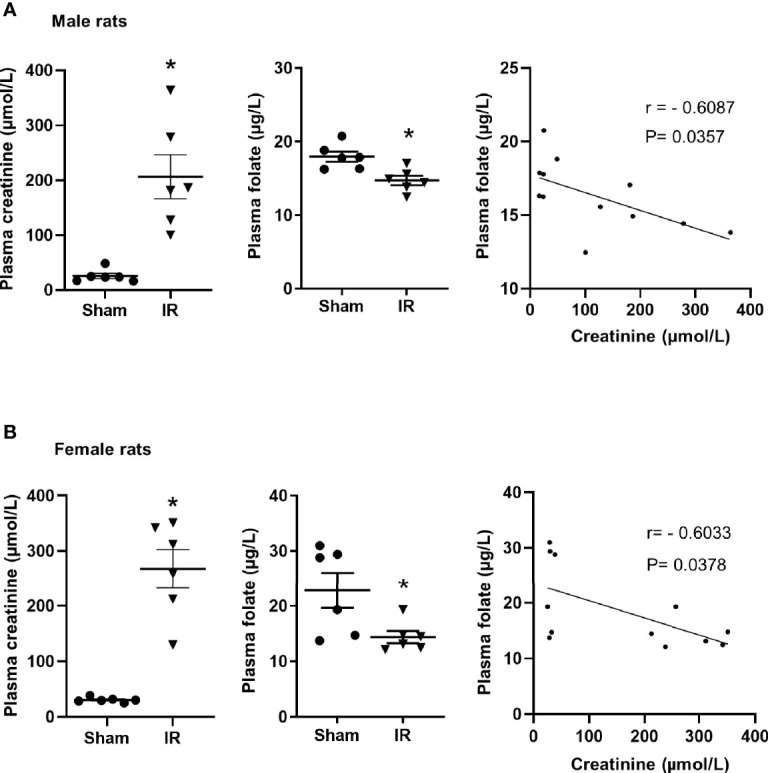
Plasma creatinine and folate measurement in rats. Rats were subjected to kidney ischemia-reperfusion (IR) or sham operation (sham). Plasma creatinine and folate were measured in **(A)** male and **(B)** female rats. Pearson’s correlation between plasma folate levels and plasma creatinine levels was analyzed. Results are expressed as mean ± SE (n = 6). *P < 0.05 when compared with the value obtained from the sham-operated group.

**Figure 2:**
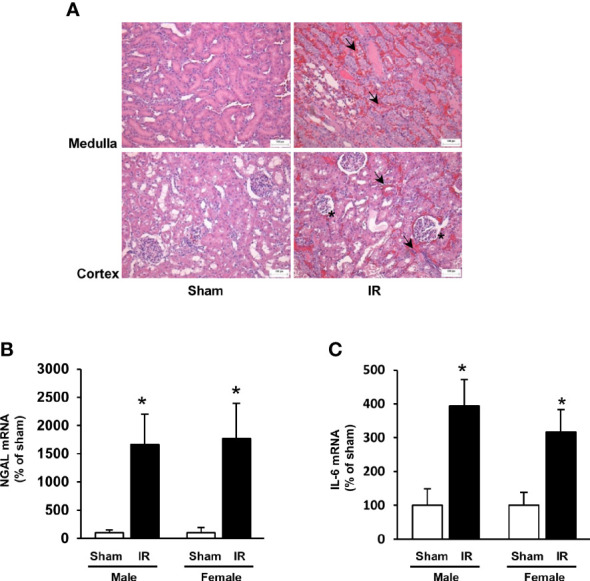
Kidney histology and expression of NGAL and IL-6. Rats were subjected to kidney ischemia-reperfusion (IR) or sham operation (sham). **(A)** Representative hematoxylin and eosin (H&E) staining images of kidney sections are shown (magnification x200). Arrows and stars point the area having red blood cell accumulation and irregular glomerular structures, respectively. The bar on the images represents 100 μm. The mRNA of NGAL **(B)** and IL-6 **(C)** was measured by real-time PCR analysis. Results are expressed as mean ± SE (n = 6). *P < 0.05 when compared with the value obtained from the sham-operated group.

Kidney ischemia-reperfusion caused a significant elevation of plasma creatinine levels in male and female rats (**Figures 1A, B**), indicating that kidney function was impaired. There was a significant decrease in plasma folate levels in rats subjected to kidney ischemia-reperfusion (**Figures 1A, B**). The plasma folate levels were inversely correlated with plasma creatinine levels in both male and female rats (**Figures 1A, B**). The histological analysis of the kidney tissue revealed irregular glomerular structure, red blood cell accumulation and tubular cell necrosis in rats subjected to ischemia-reperfusion (**Figure 2A**). There was a significant increase in the expression of NGAL and IL-6 mRNA in the kidneys of rats with ischemia-reperfusion (**Figures 2B, C**), indicating kidney injury and increased inflammatory cytokine expression.

### Ischemia-Reperfusion Decreased Folate Transporter Expression in the Rat Kidneys

**Figure 3:**
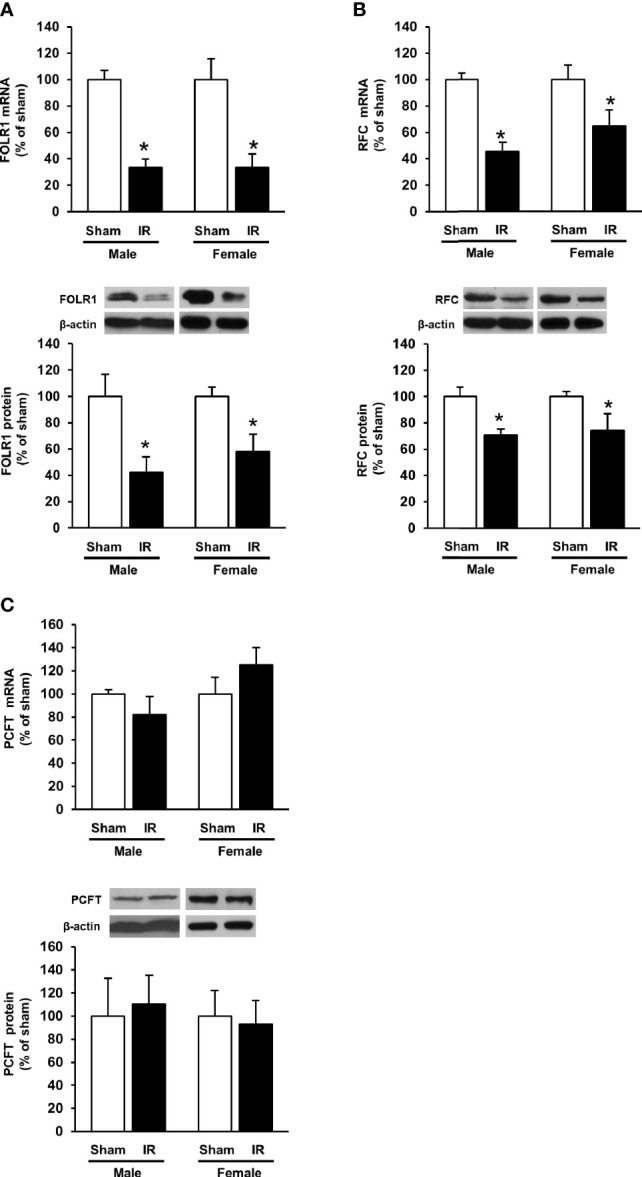
Expression of folate transporters in rat kidneys. Rats were subjected to kidney ischemia-reperfusion (IR) or sham operation (sham). The mRNA and protein of **(A)** folate receptor 1 (FOLR1), **(B)** reduced folate carrier (RFC) and **(C)** proton-coupled folate transporter (PCFT) in the kidneys of male and female rats were determined by real-time PCR and Western immunoblotting analysis, respectively. Results are expressed as mean ± SE (n = 6). *P < 0.05 when compared with the value obtained from the sham-operated group.

**Figure 4:**
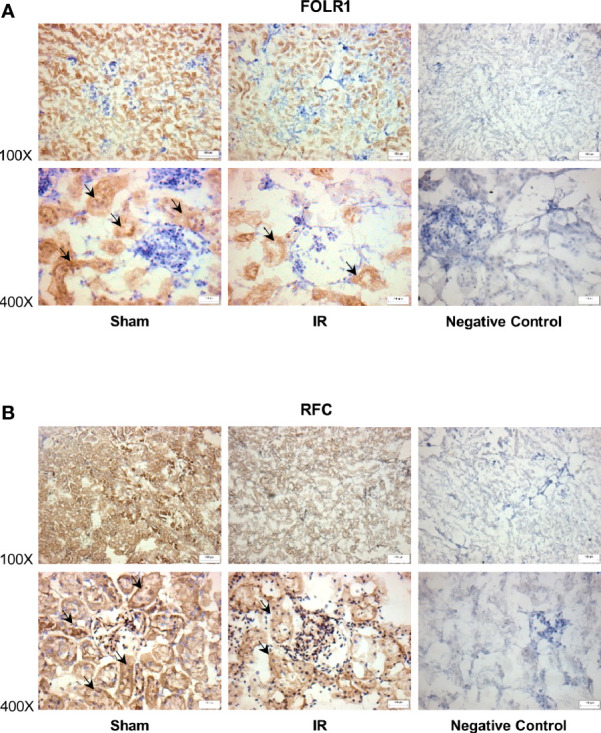
Immunohistochemical staining of folate transporters in rat kidneys. Rats were subjected to kidney ischemia-reperfusion (IR) or sham operation (sham). Immunohistochemical staining for **(A)** folate receptor 1 (FOLR1) and **(B)** reduced folate carrier (RFC) in the kidney tissue was carried out with anti-FOLR1 antibodies and anti-RFC antibodies, respectively (magnification x100 and x400). As a negative control, immunohistochemical staining was performed by using non-specific rabbit IgG as primary antibodies. Arrows point to the areas stained with FOLR1 or RFC antibodies (brown color). All staining analyses were performed in kidneys isolated from 6 rats of each group. Representative photos were shown. The bar on the image represents 100 µm.

The expression of folate transporters in the rat kidneys was examined. Ischemia-reperfusion caused a significant decrease in FOLR1 mRNA and protein levels in the kidney tissue of male and female rats (**Figure 3A**). Ischemia-reperfusion also reduced RFC mRNA and protein levels in the kidney tissue (**Figure 3B**). There was no significant change in PCFT expression upon ischemia-reperfusion (**Figure 3C**). Immunohistochemical staining of the kidneys detected FOLR1 and RFC proteins that were mainly localized in kidney tubules (**Figures 4A, B**).

### Hypoxia-Reoxygenation Decreased Folate Transporter Expression in Human Proximal Tubular Cells

**Figure 5:**
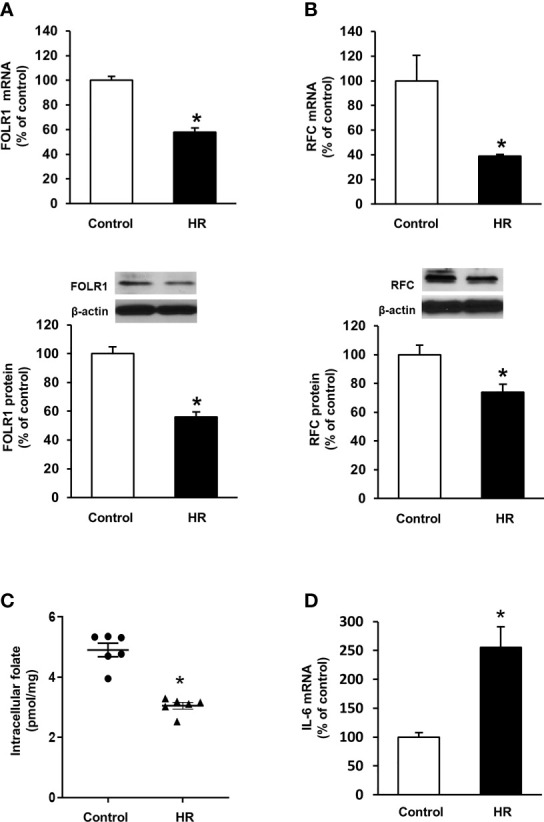
Effect of hypoxia-reoxygenation on folate transporter expression and folate levels in proximal tubular cells. Cells were subjected to 2 h hypoxia followed by reoxygenation (HR) for 48 h or 72 h. The mRNA and protein expression of **(A)** folate receptor-1 (FOLR-1) and **(B)** reduced folate carrier (RFC) were determined by real-time PCR analysis 48 h after hypoxia-reoxygenation and by Western immunoblotting analysis 72 h after hypoxia-reoxygenation, respectively. **(C)** Intracellular folate was measured 72 h after hypoxia-reoxygenation. **(D)** IL-6 mRNA was determined. Cells cultured under normal condition were used as control. Results are expressed as mean ± SE (n = 5-6). *P < 0.05 when compared with the value obtained from the control cells.

Folate reabsorption in the kidneys mainly occurs in the proximal tubules where folate transporters are abundantly expressed in tubular cells. Experiments were performed in human proximal tubular cells with hypoxia-reoxygenation that simulated ischemia-reperfusion. Hypoxia-reoxygenation caused a significant decrease in FOLR1 mRNA and protein levels in tubular cells (**Figure 5A**). Hypoxia-reoxygenation also reduced RFC expression in tubular cells (**Figure 5B**) but did not change PCFT expression (data not shown). There was a significant decrease in intracellular folate levels after hypoxia-reoxygenation (**Figure 5C**). Hypoxia-reoxygenation significantly increased IL-6 mRNA in tubular cells (**Figure 5D**).

### Reduced Sp1 Activity Might Contribute To Decreased Folate Transporter Expression During Ischemia-Reperfusion

**Figure 6:**
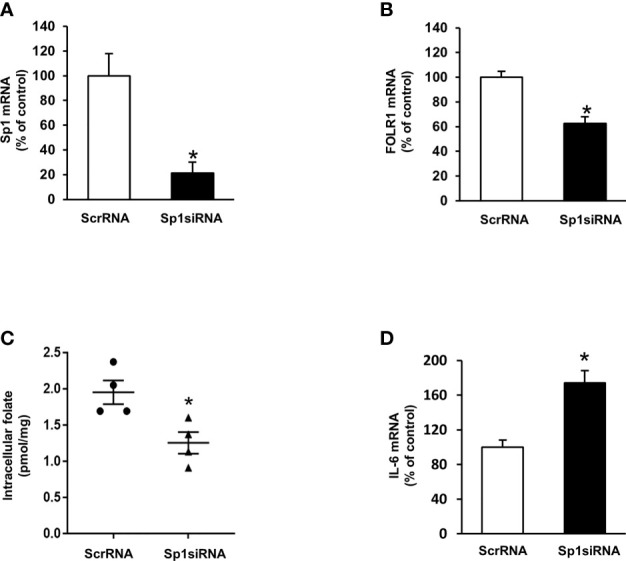
Effect of Sp1 siRNA transfection on folate receptor expression, folate content and IL-6 expression in proximal tubular cells. Cells were transfected with Sp1 siRNA or scrambled RNA (ScrRNA) as a control. **(A)** Sp1 mRNA, **(B)** folate receptor (FOLR-1) mRNA, **(C)** intracellular folate and **(D)** IL-6 mRNA was determined by real-time PCR analysis. Results are expressed as means ± SE (n = 4-6). *P < 0.05 when compared with the value obtained from cells transfected with ScrRNA.

**Figure 7:**
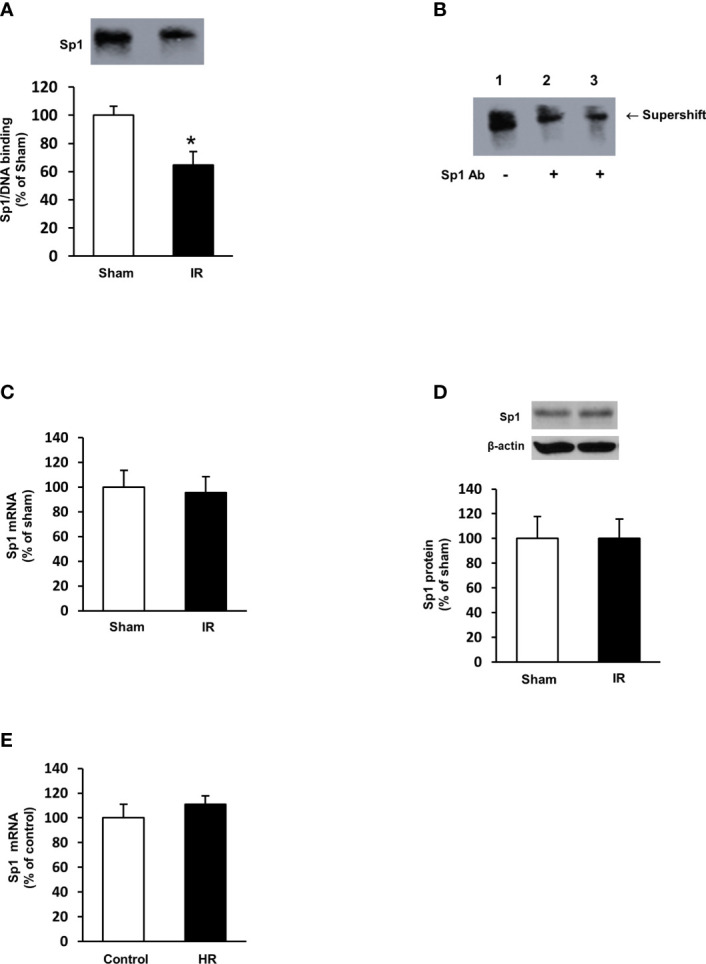
Sp1/DNA binding activity and expression in rat kidneys and proximal tubular cells. Rats were subjected to kidney ischemia-reperfusion (IR) or sham operation (sham). Nuclear proteins were isolated from the kidneys. **(A)** The DNA binding activity of Sp1 was determined by EMSA. The binding activity in the sham-operated group was expressed at 100%. **(B)** In the supershift assay, the nuclear protein and DNA oligonucleotides were incubated in the absence (lane 1) or presence of anti-Sp1 antibody (Abcam, 2 μL in lane 2 and 4 μL in lane 3) for supershift assay. The shift of the Sp1/DNA complex is indicated by an arrow. **(C)** The mRNA of Sp1 in the kidneys was determined by real-time PCR analysis. **(D)** The Sp1 protein in the kidneys was determined by Western immunoblotting analysis. **(E)** Proximal tubular cells were subjected to 2 h hypoxia followed by reoxygenation (HR) for 48 h. Cells cultured under normal condition were used as control. The mRNA of Sp1 was measured. Results are expressed as mean ± SE (n = 5-6). *P < 0.05 when compared with the value obtained from the sham-operated group or the control cells.

To investigate the mechanism by which ischemia-reperfusion decreased folate transporter expression, siRNA transfection was conducted in tubular cells. Cells were transiently transfected with Sp1 siRNA or a scrambled siRNA control. Inhibition of Sp1 expression by Sp1 siRNA transfection resulted in a significant reduction of Sp1 mRNA (**Figure 6A**) and FOLR1 mRNA expression in tubular cells (**Figure 6B**). Furthermore, silencing Sp1 caused a significant decrease in intracellular folate levels (**Figure 6C**) and a significant increase in IL-6 mRNA (**Figure 6D**). Sp1 is a transcription factor that regulates FOLR1 expression. The nuclear proteins in the kidney tissue were prepared and the EMSA was performed. Compared to the sham-operated group, there was a significant decrease in the Sp1/DNA binding activity in the kidneys collected from rats subjected to ischemia-reperfusion (**Figure 7A**). The super-shift assay was also performed to confirm the presence of Sp1 in the EMSA complex (**Figure 7B**). These results suggested that Sp1 might contribute to the down-regulation of FOLR1 expression in the kidney, which in turn, decreased folate reabsorption in tubular cells during ischemia-reperfusion injury. However, there was no significant change in Sp1 mRNA (**Figure 7C**) and protein (**Figure 7D**) levels in the kidneys upon ischemia-reperfusion. Furthermore, there was no change in Sp1 mRNA in proximal tubular cells that were subjected to hypoxia-reoxygenation (**Figure 7E**).

## Discussion

The kidneys play an important role in folate homeostasis through transporter-mediated reabsorption in proximal tubules. In the present study, ischemia-reperfusion caused severe kidney injury and a decrease in plasma folate levels in rats. Our results, for the first time, demonstrated that kidney ischemia-reperfusion significantly reduced the expression of folate transporters (FOLR1, RFC) in the kidneys of both male and female rats. Such an inhibitory effect might be mediated, in part, *via* down-regulation of transcription factor Sp1. The plasma folate levels were inversely correlated to plasma creatinine levels, suggesting that low circulating folate levels were associated with impaired kidney function.

Patients with end-stage renal disease or kidney transplantation often develop nutrient deficiency. A recent prospective study indicates that altered micronutrient status is common in patients with severe AKI regardless of receiving continuous renal replacement therapy (37). It has been reported that kidney transplant recipients have reduced blood folate levels and elevated homocysteine levels (hyperhomocysteinemia) (14), the latter is a risk factor for cardiovascular disease. Reduced serum folate and elevated homocysteine levels have also been observed in patients with chronic renal failure (11, 12). However, the mechanism of low circulating folate levels associated with kidney disease is not fully understood. In the present study, we examined the effect of AKI on plasma folate levels in a rodent model. Rats with kidney ischemia-reperfusion injury had significantly low levels of plasma folate. Further analysis revealed that the plasma folate levels inversely correlated to creatinine levels. These results suggested that as kidney function deteriorated, the peripheral circulating folate level decreased. Folate is an essential micronutrient that is required for many biochemical reactions in the body. Nutritional support is a key element for AKI patients to ensure adequate nutrient balance. According to the Kidney Disease Improving Global Outcomes (KDIGO) Clinical Practice Guideline for AKI, improving nutritional status in AKI patients is critical for better clinical outcomes (7). Our results suggest that patients with AKI may be at a higher risk of developing folate deficiency.

The 5-MTHF is the predominant form of folate in the circulation. It is estimated that 20% of circulating 5-MTHF in rats is protein-bound (17, 38). The non-protein bound folate (5-MTHF) is freely filtered in the glomeruli. Reabsorption of folate in renal proximal tubules through folate transporters is an important process to prevent urinary loss of folate. In healthy individuals, folate is reabsorbed from the lumen into tubular cells, a process primarily mediated by FOLR1 (17, 19, 39). Such a reabsorption of folate in renal proximal tubules is an important process to prevent urinary loss of folate. Diminished folate transporter expression in tubular cells might contribute to impaired folate reabsorption in the kidneys, which, in turn, led to reduced plasma (circulating) folate levels. Several lines of evidence from the present study suggested that impaired FOLR1 expression might be one of the mechanisms contributing to low circulating folate levels in AKI. Ischemia-reperfusion significantly reduced FOLR1 mRNA and protein in the kidneys. Simulation of ischemia-reperfusion in proximal tubular cells by hypoxia-reoxygenation decreased FOLR1 expression and significantly reduced intracellular folate levels. The expression of folate receptor can be regulated by a transcription factor Sp1. The human and rat folate receptor gene contains Sp1 binding sites in its promoter region (20–22). There are three Sp1 binding sites identified in human folate receptor and the binding of Sp1 to all these sites are necessary for optimal transcriptional regulation (21, 22). In the present study, ischemia-reperfusion decreased Sp1/DNA binding activity and the expression of FOLR1 in the kidney. Silencing Sp1 by siRNA transfection caused a reduction of FOLR1 expression and low folate levels in human proximal tubular cells. In a previous study, we observed that ischemia-reperfusion could reduce the phosphorylation of Sp1, which, in turn, decreased its transcriptional regulation of cystathionine-beta-synthase expression in the kidneys (30). However, it remains to be investigated whether ischemia-reperfusion alters the binding of Sp1 to the promoter region of FOLR1 in the kidneys. Aside from FOLR1-mediated folate reabsorption in the kidney, RFC is involved in transporting folate from tubular cells into the peritubular capillaries (23). Ischemia-reperfusion also inhibited RFC expression in the kidneys, which might cause less folate being transported into the circulation. Taken together, our results demonstrated that ischemia-reperfusion injury could inhibit folate transporter expression in the kidneys and reduce folate levels in the circulation.

The present study included both male and female rats. To the best of our knowledge, this is the first study reporting a downregulation of renal folate transporter expression and low plasma folate levels in a rodent model with ischemia-reperfusion induced AKI. However, the present study also had some limitations. First, down-regulation of renal folate transporter expression and low plasma folate levels were observed in rats 24 h after ischemia-reperfusion injury. It remains to be investigated whether such an injury has a long-term impact on folate homeostasis. Second, ischemia-reperfusion elicited inflammatory response in the kidneys. Folate deficiency is associated with increased oxidative stress, inflammatory response and/or hepatic steatosis in metabolic disease (40–44). Folic acid supplementation can attenuate high fat diet/fatty acid induced inflammatory cytokines including IL-6 in the liver and hepatocytes (42, 45). Our previous studies have shown that folic acid supplementation can also attenuate hyperhomocysteinemia-induced inflammatory response in the aorta (46) as well as reduce oxidative stress in rat liver and kidneys (47, 48). However, there is no direct evidence of a causal relationship between a decreased folate transporter expression/low folate levels and an increased inflammatory response in the kidneys upon ischemia-reperfusion injury. Future studies are warranted to investigate (1) whether folic acid supplementation can attenuate ischemia-reperfusion induced inflammatory cytokine expression in the kidneys; and (2) the impact of inflammation on folate transporter expression and folate status in patients with kidney injury. Third, cautions should be taken to interpret results obtained from an animal model. Results obtained from the present study need to be corroborated with clinical investigation in patients with AKI to confirm an inverse correlation between kidney function parameters and folate levels in the circulation as well as its impact on clinical outcomes. It remains to be investigated if folate supplementation can improve AKI associated biochemical and pathological changes such as inflammatory cytokine expression, oxidative stress and kidney injury. Furthermore, the present study focused on the impact of AKI on folate status and the mechanism involved. The impact of AKI on the status of other micronutrients and clinical outcomes remains to be investigated in future studies.

In conclusion, the present study has identified a significant association between kidney function and plasma folate levels. Our results, for the first time, demonstrate that kidney ischemia-reperfusion injury decreases renal folate transporter expression, in part, mediated *via* Sp1 transcriptional downregulation and causes a significant reduction of plasma folate levels in a rat model. Future studies are warranted to confirm if circulating folate level is inversely correlated with kidney function in AKI patients. Improvement of folate status in patients with kidney disease may be critical for better clinical outcomes.

## Data Availability Statement

The raw data supporting the conclusions of this article will be made available by the authors, without undue reservation.

## Ethics Statement

The animal study was reviewed and approved by University of Manitoba Protocol Management and Review Committee.

## Author Contributions

KO, YS, and TZ conceived and designed research. CW, SH, and KA-Y performed experiments and data analysis. CY, GT, and CW performed data analysis and interpretation. CY and CW prepared figures and table. CY, CW, YS, and KO drafted the manuscript. All authors edited and approved submission of the manuscript. All authors contributed to the article and approved the submitted version.

## Funding

This work was supported, in part, by grants from St. Boniface Hospital Foundation, and Natural Sciences and Engineering Research Council of Canada.

## Conflict of Interest

The authors declare that the research was conducted in the absence of any commercial or financial relationships that could be construed as a potential conflict of interest.

The handling editor declared a past co-authorship with the authors (CY and TZ).
